# Diagnostic Value of T-Cell Interferon-γ Release Assays on Cerebrospinal Fluid for Tuberculous Meningitis

**DOI:** 10.1371/journal.pone.0141814

**Published:** 2015-11-06

**Authors:** Ling Qin, Lifan Zhang, Yueqiu Zhang, Xiaochun Shi, Yao Zhang, Xiaoqing Liu

**Affiliations:** 1 Department of Infectious Diseases, Peking Union Medical College Hospital, Chinese Academy of Medical Sciences & Peking Union Medical College, Beijing, 100730, China; 2 Clinical Epidemiology Unit, Peking Union Medical College, International Clinical Epidemiology Network, Beijing, 100730, China; Chinese Academy of Medical Sciences and Peking Union Medical College, CHINA

## Abstract

Diagnosis of tuberculous meningitis (TBM) remains a challenge. This study aimed to evaluate the performance of T-SPOT.TB test on cerebrospinal fluid mononuclear cells (CSFMCs) for suspected TBM patients. 43 consecutive patients with suspected TBM were enrolled in the study from June 2011 to September 2014. T-SPOT.TB was performed on both CSFMCs and peripheral blood mononuclear cells (PBMCs). The final diagnosis of TBM was independent of the T-SPOT.TB result. The diagnostic sensitivity, specificity, predictive value, and likelihood ratio of T-SPOT.TB on CSFMCs and PBMCs were analyzed. Of the 43 patients, 12 (27.9%) were finally diagnosed with TBM, 28 (65.1%) with non-TBM, and 3 (7.0%) with indeterminate diagnoses. Of 40 cases with definite diagnoses, the sensitivity and specificity were 92.0% and 96.0% for T-SPOT.TB on CSFMCs, and 83.0% and 82.0% for T-SPOT.TB on PBMCs, respectively. The positive predictive value (PPV) and negative predictive value (NPV) of T-SPOT.TB on CSFMCs were 85.0% and 96.0%, respectively. The PPV and NPV were 67.0% and 92.0% for T-SPOT.TB on PBMCs. The difference of T-SPOT.TB between CSFMCs and PBMCs was not significant so far as sensitivity, specificity, PPV, and NPV were concerned (P>0.05 for each). However, T-SPOT.TB on CSFMC and CSFMC: PBMC in TBM cases seemed higher than that in non-TBM cases. Our study further showed that T-SPOT.TB on CSFMCs might be a rapid and accurate diagnostic test for TBM. CSFMC: PBMC T-SPOT.TB ratio might be useful for the early diagnosis of TBM.

## Introduction

Tuberculosis (TB) which is caused by *Mycobacterium tuberculosis (MTB)* primarily infects the lungs but it also affects other parts of the body. Tuberculous meningitis (TBM) is one of the most severe forms of TB. The mortality of TBM is 20–41% in the developed world, and 44–69% in developing nations[1]. Early diagnosis is challenging due to the non-specific symptoms or signs of TBM and the low sensitivity of tubercle bacilli culture in cerebrospinal fluid (CSF) [2]. Despite anti-tuberculosis treatment, TBM is still one of the main causes ofdeath and neurological sequelae as treatment given to the patients is often delayed.

The enzyme-linked immunospot assay (ELISpot) detects interferon-γ (IFN-γ) secreting T cells specific for two antigens, early secretory antigenic target-6 (ESAT-6) and culture filtrate protein-10 (CFP-10), which are present in MTB, but absent from Mycobacterium bovis BCG vaccine and most environmental mycobacteria[3]. It has been proven that T-SPOT.TB on peripheral blood samples allows accurate identification of symptom-free individuals exposed to MTB[4], but does not distinguish between latent TB infection and active TB[5].

The intracerebral inflammatory response is the main pathophysiology of TBM. IFN-γ and Antigen-stimulated T cells which produce IFN-γ play a significant role in the development of TBM[6]. Recently, at other forms of active tuberculosis, it has been shown that CFP-10 and ESAT-6 specific IFN-γ secreting T cells are concentrated. T-SPOT.TB have been reported to have diagnostic utility in pleural, ascitic, and pericardial fluid[7,8], suggesting that this assay may be a rapid, sensitive and specific marker for active TB at specific anatomical sites. However, detection of MTB-antigen-specific T cells in cerebrospinal fluid (CSF) is scientifically challenging because of the low number and short half-life of CSF T cells in TBM[9]. The sensitivity and specificity of *MTB*-specific antigen detection directly in CSF are controversial and vary in the range of 35–95% and 95–100%, respectively[9,10]. A small prospective clinical study has proven the feasibility of T-SPOT.TB on CSF for diagnosis of TBM[9].

To further determine whether the use of T-SPOT.TB on CSF samples could be a clinically accurate diagnostic method for active TBM, we performed this prospective, hospital-based study to evaluate the diagnostic sensitivity and specificity of CSF T-SPOT.TB.

## Materials and Methods

### Study populations

**Fig 1:**
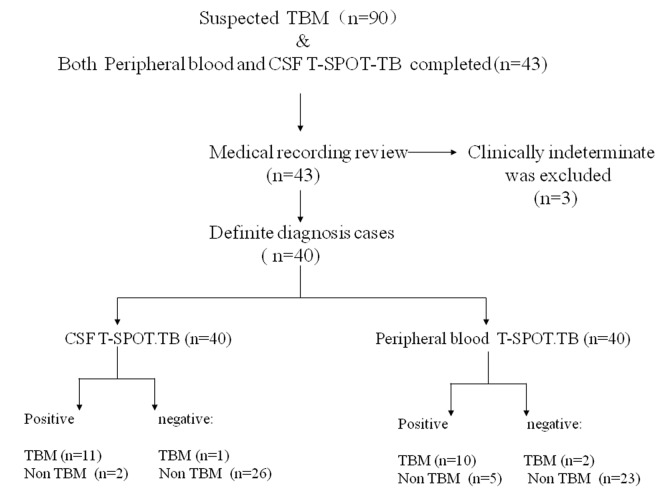
Study flow diagram.

Patients with clinical suspected TBM at Peking Union Medical College Hospital (PUMCH), Beijing were consecutively recruited between June 2011 and September 2014. The study protocol was approved by the Ethics Committee of Peking Union Medical College Hospital. Patients (>18 years) were enrolled in the study if both peripheral blood and cerebrospinal fluids (CSF) T-SPOT.TB were obtained (Fig 1). All patients provided written informed consent for the current study and the clinical study was approved by Peking Union Medical College Hospital.

Clinical information was extracted from patients’ medical recordings by researchers blinded to the T-SPOT.TB results, who also tracked patients’ treatment and discharge diagnosis. All patients had routine blood tests, including blood white blood cell (WBC), lymphocyte and HIV test. CSF was obtained by lumbar puncture and the following tests were performed: routine cell counting, routine chemistry (protein, glucose, chloride), microscopy (Gram stain, acid-fast bacilli stain and India ink stain), bacterial culture, Mycobacterium tuberculosis culture, fungal culture, TB polymerase chain reaction (PCR)(Roche Amplicor), cysticercal ELISA and a cryptococcal antigen latex agglutination test. Written informed consent for lumber puncture was routinely obtained. In patients with abnormal mental state, consent was obtained from a first-degree relative. Heparinized samples of venous blood (4 ml) and of CSF (4 ml) were obtained and processed for detecting specific T cell responses to RD1 encoded antigens by T-SPOT.TB (Oxford Immunotec, Abingdon, UK).

### Diagnosis of TBM

According to predefined criteria used in previous studies[9,11], patients were diagnosed as definite TBM (blood culture, CSF culture or PCR positive for tuberculosis), highly probable TBM (presence of clinical features of an aseptic bacterial meningitis, negative test for other causes, two of the following (chest X-ray consistent with active TB, a CT or MRI consistent with TBM such as hydrocephalus or basal enhancement), and appropriate response to anti-tuberculosis therapy), clinically indeterminate (neither high probable or reliably excluded) or non-TBM (an alternate definite cause for meningitis identified and response to appropriate non-TB therapy).

### CSF and peripheral blood Lymphocyte RD1 Antigen-specific IFN-γ Responses

4 ml of CSF were collected from each patient and was performed within 6 hours after collection by laboratory personnel blinded to patients’ clinical data. T-SPOT.TB utilized AIM-V (GIBCOTM AIM V Medium liquid, Invitrogen, US) as negative control, PHA as positive control, and ESAT-6 and CFP-10 as specific antigens, respectively. CSF mononuclear cells (CSFMCs) were separated by Ficoil-Hypaque gradient centrifugation and plated (2.5×10^5^ per well) on a plate pre-coated with anantibody against interferon-**γ**. After incubation 16–18 h at 37°C in 5% carbon dioxide, plate wells were washed and incubated with a conjugate against the antibody used and an enzyme substrate. Spot-forming cells (SFCs) that represented antigen-specific T cells secreting interferon-**γ** were counted with an automated ELISPOT reader (AID-ispot, Strassberg, Germany). A positive response was defined as 6 or more SFCs in the target well. The background number of spots in the negative control well for CSFMCs should be less than 10 spots.When the cell counts in CSF was not possible to harvest 2.5x10^5^ cells per well, we used the ratio between 2.5x10^5^, the target number and the actually number to adjust the result. 4 ml of peripheral blood were also collected from each patient and RD-1 ELISPOT assay protocol for peripheral blood mononuclear cells (PBMCs) was same with that for CSFMCs.

### Statistical and Data Analysis

Sensitivity, specificity, positive predictive value (PPV), negative predictive value (NPV), likelihood ratio positive (LR+), and likelihood ratio negative (LR-) were calculated to evaluate diagnostic performance of T-SPOT.TB on CSFMCs and PBMCs. Means were used for data of normal distribution, while median and IQR were used for data that were not normally distributed. Means and medians were compared using student’s t-test or Wilcoxon test as appropriate. Positive proportions were compared using Pearson’s Chi-square test. 95% confidence intervals (CI) were estimated according to the binomial distribution. Significance was inferred for P<0.05 and statistical analysis was performed by SPSS16.0 (SPSS Inc, Chicago, IL).

## Results

Forty-three HIV negative Chinese patients with suspected TBM were recruited in our study. Four were diagnosed with definite TBM, eight with highly probable TBM, twenty-eight with non-TBM, and three patients with clinically indeterminate TBM. After excluding three patients with clinically indeterminate TBM, the demographic and clinical characteristics of forty patients were showed in Table 1. Table 2 summarizes the characteristics of twelve patients with TBM. Of four patients with definite TBM, two were confirmed by CSF culture, one by positive anti-fast stain, and another one by positive PCR. Of twenty-eight non-TBM cases, nine were diagnosed with virus meningitis, five with neuromyelitis optica, four with non-tuberculous pyogenic meningitis, three with cryptococcus meningitis, two with brucellosis, two with SLE, one with cysticercus, one with acute myeloid leukemia, and one with lymphoma. All the cases were microbiologically or histologically confirmed and recovered without any anti-tuberculous therapy.

**Table 1:** Demographic and clinical characteristics of the patients.

Characteristic	TBM	Non-TBM
Case number	12	28
Age in years (median, IQR)	46[24–59]	43[29–55]
Sex		
Male (%)	6(50.0%)	16(57.1%)
Female (%)	6(50.0%)	12(42.9%)
Duration in days (median, IQR)	22[11–75]	30[14–150]
Evidences of previous TB (%)	0	2(7.1%)
Pre-existing conditions (%)	4(33.3%)	2(7.1%)
Blood tests (median, IQR)		
Lymphocytes (10^9/L)	0.99[0.57–1.42]	1.46[1.12–1.99]
ESR(mm/h)	45[15–68]	15[5–36]
CRP(mg/L)	7.64[2.31–35.02]	1.09[0.29–11.84]
CSF tests (median, IQR)		
Pressure(mmH2O)	205[130–325]	190[130–258]
Mononuclear cells(cells/ul)	139[91–305]	84[30–131]
Glu(mmol/L)	1.4[0.9–2.5]	3.0[2.2–3.4]
Cl(mmol/L)	112.5[107.3–118.0]	118.5[110.5–122.5]

Duration: the course of the disease before definitive diagnosis was made

Pre-existing conditions: diseases could damage the function of immune system.

**Table 2:** Clinical characteristics of tuberculosis meningitis cases (n = 12).

Case No.	Age/gender	Basic disease	Steroids use	Fever	Clinical features	Duration (days)	Blood lymphocyte (10^9/L)	Brain MRI or CT	Lung TB	Other site TB	Response	Diagnosis method	T-SPOT.TB (SFCs/10^6MC)	
													PBMC	CSFMC
1	49/M	None	no	38.9	headache	14	1.11	occipital nodular, meningeal enhancement	yes	no	better	CSF anti-fast Stain +	284	104
2	43/M	DM	no	39	headache, septic shock	120	0.86	occipital meningeal enhancement	yes	no	better	HP	0	0
3	60/M	None	no	39.2	headache	30	2.05	meningeal enhancement	yes	no	better	HP	24	268
4	21/F	None	no	39	headache, confusion	90	1.45	intracranial multiple nodular	yes	no	better	HP	2548	140
5	39/F	DM	no	38.6	headache, faint, seizure	7	1.14	hydrocephalus	yes	no	better	HP	960	4500
6	30/F	None	no	39	headache	7	1.32	intracranial multiple nodular, meningeal enhancement	no	no	better	HP	56	5248
7	75/F	HTN	no	39	headache, faint	360	0.58	multiple intracranial nodular	yes	SPINE	better	HP	740	156
8	22/M	None	no	39	confusion	14	0.53	meningeal enhancement, ventriculomegaly	yes	no	better	CSF TB Culture +	1472	1212
9	74/F	HTN	no	39.5	headache	30	0.56	meningeal enhancement	yes	no	better	PCR+	2068	264
10	20/F	SLE	yes	41	headache	30	0.52	meningeal enhancement ventriculomegaly	yes	no	die	Blood TB Culture +	0	68
11	57/M	DM	no	38	headache	10	1.74	frontal nodule, meningeal enhancement	yes	no	better	HP	756	512
12	53/M	COPD	no	39	confusion, triplegia	14	0.81	multiple intracranial nodular, meningeal enhancement	yes	no	better	HP	76	300

DM: diabetes mellitus; HTN: hypertension; SLE: systemic lupus erythematosus;COPD: chronic obstructive pulmonary disease; TB: tuberculosis; HP: highly probable; PBMC: peripheral blood mononuclear cell; CSF: cerebrospinal fluid

### Sensitivity and Specificity of T-SPOT.TB on CSFMCs and PBMCs

In Table 3, The T-SPOT.TB test was positive on CSFMCs in 11 of 12 TBM cases, giving a sensitivity of 92% (95%CI [62%, 100%]). The T-SPOT.TB test was positive on PBMCs in 10 of 12 TBM cases, giving a sensitivity of 83% (95%CI [52%, 98%]). There was no significant difference in T-SPOT.TB sensitivities between CSFMCs and PBMCs (p = 1.000). One patient (Case 2) with diabetes mellitus (DM) who might suffer from hematogenously spread TB showed negative ELISpot results on both PBMCs and CSFMCs. T-SPOT.TB result of the patient (Case 10) with systemic lupus erythematosus (SLE) who received alarge dose of steroids and immunosuppressant showed negative T-SPOT.TB on PBMCs but positive on CSFMCs.

**Table 3:** Single and combined diagnostic parameters of T-SPOT.TB on CSFMC and PBMC.

T-SPOT.TB on	Sensitivity (95%CI)	Specificity (95%CI)	PPV (95%CI)	NPV (95%CI)	LR+ (95%CI)	LR- (95%CI)
CSFMC	0.92 (0.62–1.00)	0.93 (0.76–0.99)	0.85 (0.55–0.98)	0.96 (0.81–1.00)	12.83 (3.34–49.32)	0.09 (0.01–0.59)
PBMC	0.83 (0.52–0.98)	0.82 (0.63–0.94)	0.67 (0.38–0.88)	0.92 (0.74–0.99)	4.67 (2.03–10.74)	0.20 (0.06–0.73)
PBMC or CSFMC	0.92 (0.62–1.00)	0.82 (0.63–0.94)	0.69 (0.41–0.89)	0.96 (0.79–1.00)	5.13 (2.28–11.57)	0.10 (0.02–0.67)
PBMC and CSFMC	0.83 (0.52–0.98)	0.93 (0.76–0.99)	0.83 (0.52–0.98)	0.93 (0.76–0.99)	11.67 (3.00–45.42)	0.18 (0.05–0.64)

T-SPOT.TB on CSFMCs was negative in 26 non-TBM cases, giving a specificity of 93% (95%CI [76%, 99%]). T-SPOT.TB on PBMCs was negative in 23 non-TBM cases, giving a specificity of 82% (95%CI [63%, 94%]). There were no significantly different T-SPOT.TB specificities between CSFMCs and PBMCs (P = 0.25). Of 2 non-TBM cases with positive T-SPOT.TB results on both CSFMCs and PBMCs, one was diagnosed with virus meningitis and the other with brucellosis. The SFCs were 36 and 24 per million monocytes on CSFMCs, and 168 and 476 per million monocytes on PBMCs, respectively. Of the other three non-TBM patients with positive T-SPOT.TB results only on PBMCs, one was diagnosed with virus meningitis, one with cryptococcus meningitis, and one with neuromyelitisoptica. The SFCs were 112, 88 and 500 per million monocytes on PBMCs. Of five non-TBM cases with positive T-SPOT.TB results on PBMCs, previous history of pulmonary TB was revealed in two patients with virus meningitis (Table 4).

**Table 4:** CSFMC: PBMC ratio in TBM or Non-TBM cases with positive T-SPOT.TB results.

T-SPOT.TB Positive	Frequencies of SFCs/10^6^MC in (median, IQR)		
	PBMC	CSFMC	CSFMC:PBMC
TBM	284	104	0.37
	24	268	11.17
	2548	140	0.05
	960	4500	4.69
	56	5248	93.71
	740	156	0.21
	1472	1212	0.82
	2068	264	0.13
	0	68	∞
	756	512	0.68
	76	300	3.95
Median (n = 11)	740	264	0.82
Non-TBM	168	36	0.21
	476	24	0.05
	112	0	0
	88	0	0
	500	0	0
Median(n = 5)	168	0	0

### Predictive Value and Likelihood Ratio of T-SPOT.TB on CSFMCs and PBMCs

We also analyzed the predictive values (PV) and likelihood ratios (LR) of T-SPOT.TB in 40 patients with definitive diagnosis (Table 3). Of 13 patients with positive T-SPOT.TB results on CSFMCs, 11 were diagnosed with TBM, giving the positive predictive value (PPV) of 85.0%. Of 15 patients with positive T- SPOT.TB results on PBMCs, 10 were diagnosed with TBM, giving the positive predictive value of 67%. The difference between them was not significant (P = 0.396). Of 27 cases with negative T-SPOT.TB results on CSFMCs, 26 were diagnosed with non-TBM, giving the negative predictive value (NPV) of 96%. Of 25 cases with negative T- SPOT.TB results on PBMCs, 23 were diagnosed with non-TBM, giving the negative predictive value of 92%. The difference between them was not significant (P = 0.945). The positive likelihood ratio (PLR) and negative likelihood ratio (NLR) of T-SPOT.TB on CSFMCs were 12.83 and 0.09, respectively. The PLR and NLR of T-SPOT.TB on PBMCs were 4.67 and 0.20, respectively. Both the PLR and NLR of T-SPOT.TB on CSFMCs were better than that on PBMCs.

Compared with T-SPOT.TB on CSFMCs alone, the combination of T-SPOT.TB on CSFMCs and PBMCs appeared no advantage, but increased the specificity of T-SPOT.TB on PBMCs alone from 82% up to 93% when combined, and increased PLR from 4.67 up to 11.67 (Table 3).

### Comparison of Frequencies of RD1 antigen-specific IFN-γ secreting T cells on CSF and peripheral blood in TBM patients

The frequencies of cells responding to ESAT-6 and CFP-10 in CSF from TBM patients were not significantly different from those observed in the blood (Table 5, p = 0.728 for ESAT-6, p = 0.944 for CFP-10, and p = 0.833 for total). We also summarized CSFMC: PBMC T-SPOT.TB ratio for patients with positive T-SPOT.TB results (Table 4). In our study, CSFMC: PBMC T-SPOT.TB ratio >1.0 were found in 5 cases of 12 definite TBM and the median CSFMC: PBMC T-SPOT.TB ratio was 0.82. In non-TBM cases, the ratios of CSFMC: PBMC T-SPOT.TB in 5 patients with positive T-SPOT.TB on CSFMCs and/or PBMCs, were all less than 1.0.

**Table 5:** Frequencies of MTB-specific IFN-γ secreting T cells on CSFMCs and PBMCs.

	F requencies of SFCs/10^6^MC in (median, IQR)		
	PBMC	CSFMC	P value
ESAT-6	400[184–616]	148[51–1880]	0.728
CFP-10	310[48–891]	196[68–296]	0.944
T-SPOT.*TB*	748[71–1621]	268[140–1212]	0.833

## Discussion

In this study, we found that T-SPOT.TB on CSFMC is a useful diagnostic test for assessment of patients with suspected TBM, with higher sensitivity (92%, 95% CI [62%, 100%]) and specificity (93%,95%CI [76%, 99%]) than traditional standard diagnostic tests. In another pilot study, ELISpot was also proven to detect MTB-antigen-specific T cells in CSF with a high diagnostic sensitivity of 90% (95%CI [56%, 100%]) and specificity of 100% (95%CI [59%, 100%])[9].Among 12 TBM cases in our study, only 1 (8.3%) was positive by Ziehl-Neelsen staining, 1 (8.3%) by positive CSF culture, and 1 by positive PCR. As we know, the sensitivity of Ziehl-Neelsen staining in detecting acid-fast bacilli in CSF is generally low and ranges from 10% to 60%[2]. Moreover, a large volume of CSF is required for a more sensitive result but is difficult to obtain in patients with a low total CSF volume. Besides, the sensitivity of CSF culture to detect MTB is also low and ranges from 40% to 60% as culture is less sensitive inpaucibacillary conditions[3]. Although studies show the sensitivity of PCR assays for TBM ranges from 31% to 100% and specificity from 66% to 100%[12], the paucibacillary nature and presence of amplification inhibitor in CSF specimens are the main challenges to applying the PCR method to detect MTB.

Plenty of activated CSFMCs and PBMCs to TB are necessary for successful T-SPOT.TB detection. Limited lymphocyte counts orimpaired cell-mediated immunity account for the false negativeCSF T-SPOT.TB results in TBM cases (lower sensitivity) [13]. HIV-infected patients were not enrolled in our study for decreased CD4 lymphocytes in CSF and blood, which might increase the ratio of false negatives. Serious TB infection might account for a false negative T-SPOT.TB result in either CSFMCs or PBMCs due to decreased allergic ability of lymphocytes in CSF and blood, as has been reported[14]. In our study, case 2 with DM is diagnosed as serious TBM with septic shock, syncope, and suspected blood stream TB infection on admission. The negative T-SPOT.TB result on both CSFMC and PBMC may be due to the failed lymphocyte compartmentalization, migration and activation to RD1 peptides[9]. Case 10 diagnosed with TBM was a SLE patient with the history of high-dose steroid and immunosuppressant usage. Although blood TB culture and T-SPOT.TB on CSFMCs were positive, T-SPOT.TB on PBMCs was negative. A CSFMC: PBMC T-SPOT.TB ratio >1.0 is reported to indicate a high possibility of TBM[15]. In this study, CSFMC: PBMC T-SPOT.TB ratio with definite TBM was higher than that of non-TBM patients with positive T-SPOT.TB. CSFMC: PBMC T-SPOT.TB ratio might be useful for early diagnosis of TBM. The history of drug administration might account for the negative T-SPOT.TB result on PBMC; however, the mechanism of positive T-SPOT.TB on CSFMC with negative result on PBMC is unclear.

A prior history of TB might decrease the specificity of T-SPOT.TB on PBMCs compared to CSFMCs. Of 5 non-TBM cases with positive T-SPOT.TB on PBMCs, 2 harbored an old TB history. 2 of the other 3 patients also had positive results on CSFMC, despite low frequencies of SFCs and CSFMC: PBMC T-SPOT.TB ratio.

The predictive value and likelihood ratio of T-SPOT.TB on CSFMCs was higher than that on PBMCs. The NPV was 96% and NLR was 0.09 for T-SPOT.TB on CSFMCs, indicating high sensitivity and specificity of the test in CSF. We further evaluated thediagnostic performance of T-SPOT.TB on CSFMCs and/or PBMCs. Combining the tests showed lower sensitivity and specificity than the T-SPOT.TB test on CSFMCs alone, thus we were unable to improve diagnostic efficiency with the combined results. However, the T-SPOT.TB ratio on CSFMC: PBMC was supportive of a TBM diagnosis.

## Conclusion

Comparing with peripheral blood ELISpot, we have demonstrated that CSF ELISpot could be considered as a potential new diagnostic test for TBM, which is rapid, practicable and appears to have high sensitivity and specificity. The T-SPOT.TB ratio on CSFMC: PBMC might be a new approach to making a TBM diagnosis. However, the microbiological confirmation cases were fewer and our small case-control studies may tend to overestimate the accuracy of the test. Large prospective studies are now required to validate the clinical utility of CSF ELISpot in routine practice for diagnosis of TBM.

## Study Limitation

The primary limitation of this study is the small case control study design. Typical biases exist, and the statistical analysis may be limited given the small number of cases. So the sensitivity and specificity might be underestimated or overestimated. Another significant weakness of our study is the lack of microbiological confirmation of TB, despite these limitations, this study reported some important findings and convincible conclusions in the resource-poor setting.
